# Development and Validation of Real-Time PCR for Rapid Detection of *Mecistocirrus digitatus*

**DOI:** 10.1371/journal.pone.0063019

**Published:** 2013-04-30

**Authors:** Subhra Subhadra, Mohanraj Karthik, Muthusamy Raman

**Affiliations:** Department of Veterinary Parasitology, Madras Veterinary College, Tamil Nadu Veterinary and Animal Sciences University, Chennai, Tamil Nadu, India; University of Georgia, United States of America

## Abstract

Hematophagous activity of *Mecistocirrus digitatus*, which causes substantial blood and weight loss in large ruminants, is an emerging challenge due to the economic loss it brings to the livestock industry. Infected animals are treated with anthelmintic drugs, based on the identification of helminth species and the severity of infection; however, traditional methods such as microscopic identification and the counting of eggs for diagnosis and determination of level of infection are laborious, cumbersome and unreliable. To facilitate the detection of this parasite, a SYBR green-based real-time PCR was standardized and validated for the detection of *M. digitatus* infection in cattle and buffaloes. Oligonucleotides were designed to amplify partial Internal Transcribed Spacer (ITS)-1 sequence of *M. digitatus.* The specificity of the primers was confirmed by non-amplification of DNA extracted from other commonly occurring gastrointestinal nematodes in ruminants. Plasmids were ligated with partial ITS-1 sequence of *M. digitatus*, serially diluted (hundred fold) and used as standards in the real-time PCR assay. The quantification cycle (Cq) values were plotted against the standard DNA concentration to produce a standard curve. The assay was sensitive enough to detect one plasmid containing the *M. digitatus* DNA. Clinical application of this assay was validated by testing the DNA extracted from the faeces of naturally infected cattle (n = 40) and buffaloes (n = 25). The results were compared with our standard curve to calculate the quantity of *M. digitatus* in each faecal sample. The Cq value of the assay depicted a strong linear relationship with faecal DNA content, with a regression coefficient of 0.984 and efficiency of 99%. This assay has noteworthy advantages over the conventional methods of diagnosis because it is more specific, sensitive and reliable.

## Introduction

Sub-clinical parasitism due to gastrointestinal nematodes (GINs) is a major problem in ruminant livestock because it leads to huge financial losses. Among the GINs affecting large ruminants, *Mecistocirrus digitatus*, commonly known as a large stomach worm, is a bloodsucking helminth found in the abomasum of Asian zebu cattle (*Bos indicus*) and buffalo (*Bubalis bubalis*) [1]. In the abomasum of infected animals, this nematode is reported to cause severe micro- and macroscopic lesions such as mucosal inflammation, haemorrhage, ulcers and necrosis [2]. Occurrence of *M. digitatus* was recorded as early as the 1920s, from the abomasum of cattle in India [3], [4]. Various studies have confirmed the presence of *M. digitatus* infection in cattle and buffaloes in different parts of India [5], [6], [7], [8]. *M. digitatus* may be found in mixed infection in ruminants along with other GINs such as *Ostertagia ostertagi*, *Trichostrongylus spp.*, *Haemonchus placei*, *Cooperia spp.*, etc., which makes the eradication of GINs a difficult task using chemotherapeutic agents [9]. In spite of the studies on the biology [10] and geographical distribution of this parasite [11], [12], [13], there is a dearth of information on detection and control strategies. Furthermore, the longer pre-patent period (up to 60 days) of this bloodsucking GIN [14] affects early detection.

The diagnosis of this bloodsucking nematode is done by identification of eggs in the faeces of infected animals and infective L3 larvae after coproculture. Given the fact that the average length of *M. digitatus* egg (95–122 µm) [10], [11] is either the same as or slightly larger than other trichostrongylid nematode eggs, the conventional morphometric and microscopic examination fails to differentiate between trichostrongyle eggs. This problem calls for a more sensitive and reliable assay to provide information on the preponderance and relative abundance of this nematode. Molecular techniques are extensively used for sensitive and specific detection of common GINs, including *M. digitatus* [15], [16], [17], [18]. In 2002, Samson-Himmestjerna *et al*. [19] developed the TaqMan based qPCR assay to detect and quantify common GINs such as *Haemonchus contortus*, *Ostertagia leptospicularis*, *Trichostrongylus colubriformis* and *Cooperia curticei* based on ITS-2 sequence of ribosomal DNA (rDNA). Later, Bott *et al.* [20] developed a SYBR green-based real-time assay for the detection of strongylids such as *H. contortus*, *Teladorsagia circumcincta*, *Trichostrongylus spp.*, *Cooperia oncophora*, *Oesophagostomum columbianum* and *Oesophagostomum venulosum*. More recently, during 2009, the TaqMan based real-time assay was developed and validated for the detection of *H. contortus* and *T. circumcincta* [21]. The aim of the present study was to standardize a SYBR green-based real-time PCR for the detection and quantification of *M. digitatus* infection and validate the assay in naturally infected large ruminants such as non-descript cattle (NDC), cross-bred jersey cattle (CBJC), cross-bred Holstein Friesian cattle (CBHFC), non-descript buffaloes (NDB) and graded Murrah buffaloes (GMB).

## Materials and Methods

### Ethics Statement

Permission was obtained from the chief health officer of Chennai Corporation to collect adult worms from the abomasum of cattle (NDC, CBJC and CBHFC) and buffaloes (NDB and GMB) for research purpose at the corporation’s slaughter house in Perambur. The collection of faecal samples for this work was conducted in accordance with the guidelines and approval of the Institutional Animal Ethical Committee of the Tamil Nadu Veterinary and Animal Sciences University in Chennai, India (approval#318/DFBS/IAEC/2010).

### Extraction of gDNA from Adult *M. digitatus* Worms

Adult *M. digitatus* worms (n = 25) were collected from the abomasum of cattle (n = 15) and buffaloes (n = 10) at the Chennai Corporation slaughter house in Perambur. The worms were individually identified, on the basis of copulatory bursa and spicules, by light microscopy [5] and the keys provided by Whitlock (1960) [22]. The DNA was extracted, as described by Sambrook and Russell (2001) [23], with minor modifications. The purified DNA was eluted with TE buffer and stored at −20°C until being used as a positive control.

### Extraction of gDNA from Faecal Samples

Faecal samples (n = 65) were collected, i.e., 40 samples from cattle (NDC-23 samples, CBJC-7 samples and CBHFC-10 samples) and 25 samples from buffaloes (NDB-18 samples and GMB-7 samples) showing symptoms of diarrhoea from villages across the districts of Chennai, Tanjore, Tiruchirappalli, Thiruvarur, Ernakulam, Vijayawada, Nellore, Nagapattinam and Kodaikanal in Southern India. The faecal samples were transported under cold condition to the laboratory. The DNA was extracted by using QIAmp stool DNA kit (Qiagen, Germany) as per the manufacturer’s protocol. The PCR inhibitors present in the faecal samples were removed by the use of InhibitEX tablet provided in the kit in order to increase the sensitivity of the subsequent PCR assays. The purified DNA was stored in TE buffer at –20°C till use.

### Primer Design

A pair of oligonucleotides was designed to amplify part of the ITS-1 region of *M. digitatus* rDNA. The forward and reverse primers were chosen manually from sequences available in NCBI, GenBank (GenBank AB222059.1). The primers are MD ITSF (5′-TCACTTTGATTACGAGAATCCAACAG) and MD ITSR (5′- GTCTAAATCTCAACTATCATTAAACGTGA).

### Standardization of Conventional PCR

The purified *M. digitatus* gDNA was subjected to gradient PCR (55–65°C), using Taq DNA polymerase 2X Master Mix Red (containing 0.2 units/µl Ampliqon Taq DNA polymerase, 0.4 mM dNTPs and 3.0 mM MgCl_2_) (Ampliqon, Bie & Berntsen, Herlev, Denmark) and 10 picomole of each forward and reverse primer (Eurofins MWG Operon, Germany). PCR cycling conditions were as follows: one cycle of initial denaturation at 94°C for 5 minutes; followed by 40 cycles of (94°C, 55–65°C and 72°C for 30 seconds each) followed by final extension step of 72°C for 10 minutes. No-template control (NTC) and negative amplification control (NAC) were also included with each run. The specificity of the primers was checked with DNA extracted from eight other nematodes viz., *H. contortus*, *Trichostrongylus spp.*, *O. ostertagi*, *Oesophagostomum radiatum*, *Cooperia spp*., *Nematodirus spp.*, *Bunostomum spp.* and *Trichuris spp*., which are prevalent in this geographical location. The PCR product was electrophoresed through 2% agarose gel to check for positive PCR amplification.

### Purification, Cloning and Sequencing of PCR Product

The desired PCR product was purified using QIAQuick PCR purification kit (Qiagen, Hilden, Germany). The purified product was ligated into plasmid (pTZ57R/T) and transferred into *E. coli* DH5α cells using InsTAclone PCR cloning kit (Fermentas, European Union). Plasmid DNA was purified using Fast Plasmid mini-kit (Eppendorf, Germany) and was sequenced on both strands using the Big Dye Terminator v. 1.1 Cycle sequencing kit (Applied Biosystems, Foster City, CA) on a PRISM 3100 Genetic Analyzer.

### Standard DNA for Real-time PCR

Recombinant plasmids containing partial ITS-1 sequence of *M. digitatus* were quantified using Biophotometer plus (Eppendorf, Germany) and were used as standards for real-time PCR assay. Serial hundred fold dilutions of the plasmid representing 1.5×10^8^ to 1.5×10^–6^ plasmid molecules/µl were made and aliquots of each dilution were stored at −20°C.

### Standardization of Real-time PCR for Detection of *M. digitatus*

Real-time PCR assay was performed in Bio-Rad CFX-96 real-time PCR machine (Bio-Rad Laboratories, Hercules, CA) and the data generated were analyzed with the CFX Manager™ Software (version 2.0). The assay was carried out in a total reaction volume of 10 µl that consisted of 5.0 µl of 2X SsoAdvanced SYBR green super mix (containing dNTPs, Sso7d fusion polymerase, MgCl_2_, SYBR green I)(Bio-Rad Laboratories, Hercules, CA), 5.0 picomoles of each primer (Eurofins MWG Operon, Germany) and 1.0 µl of DNA template. All the samples, including the standards, NTC and NAC, were run in triplicates and all experiments were repeated at least twice. The protocol was as follows: 5 minutes at 95°C, 40 cycles at 95°C for 5 seconds, 58°C for 20 seconds and 72°C for 10 seconds. Amplicons were subjected to melt curve analysis by increasing the temperature from 58°C to 95°C at an increment of 0.5°C per second.

### Testing of Faecal Samples Collected from Naturally Infected Cattle and Buffaloes

Triplicates of DNA extracted from faecal samples collected from naturally infected cattle (n = 40) and buffaloes (n = 25) were used to detect and quantify *M. digitatus* infection by standardized real-time PCR assay as described above. The mean Cq values were compared with the standard curve data to calculate the quantity of DNA in each sample.

## Results

### Standardization of Conventional PCR

**Figure 1:**
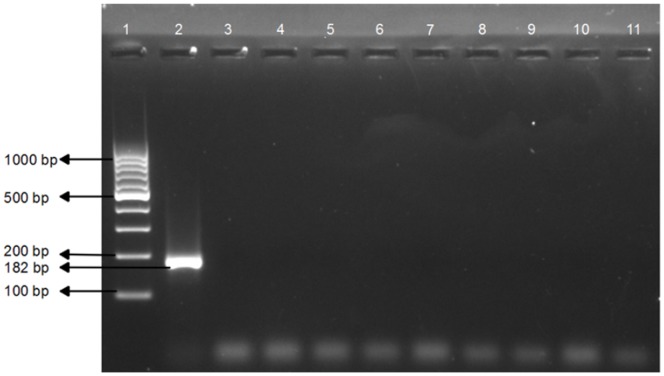
Species-specific PCR for detection of *M.* *digitatus*. Lane 1∶100–1000 bp DNA ladder, Fermentas Lane 2: *Mecistocirrus digitatus* DNA (182 bp) Lane 3: *Haemonchus contortus* DNA Lane 4: *Trichostrongylus spp.* DNA Lane 5: *Ostertagia ostertagi* DNA Lane 6: *Oesophagostomum radiatum* DNA Lane 7: *Cooperia spp*. DNA Lane 8: *Nematodirus spp.* DNA Lane 9: *Trichuris spp*. DNA Lane 10: *Bunostomum spp.* DNA Lane 11: No Template Control.

**Figure 2:**
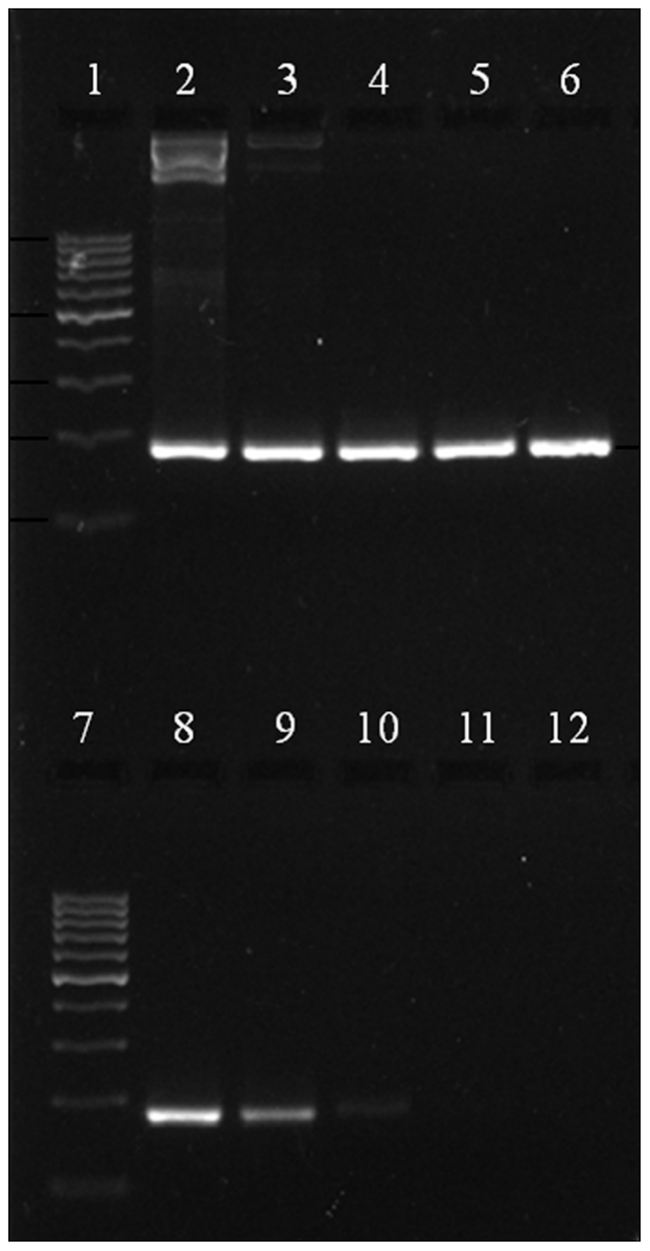
Sensitivity PCR to detect quantity of *M.* *digitatus* DNA. Lane 1&7∶100–1000 bp DNA ladder, Fermentas Lane 2–11: Serial 10 fold dilution of plasmid DNA (1.5×10^8^ to 1.5×10^0^ molecules/µl) Lane 12: No Template Control.

Specific amplification of 182 bp was observed with the positive control at 58°C and 1.5 mM MgCl_2_, while the NTC did not show any amplification. No amplification was observed with DNA extracted from eight other nematode species (Fig. 1). The PCR detected as low as 1.5×10^2^ plasmid molecules/µl of DNA (Fig. 2). The newly designed primers were found to be both species specific and sensitive. A BLAST search of the sequence result was carried out to confirm the identity of the sequence, which revealed a 98% identity with the existing *M. digitatus* sequences in GenBank, NCBI, thus confirming the identity of the species. The sequence was submitted in the EMBL-EBI database under the accession number HE974385.

### Standardization of Real-time PCR for Detection of *M. digitatus*

**Figure 3:**
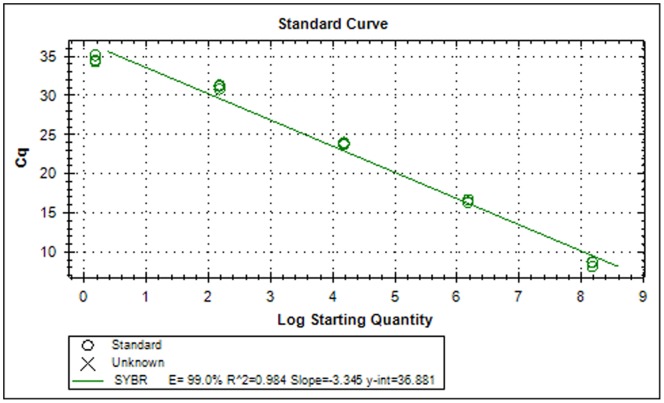
Standard curve of serial 100 fold dilutions of the plasmid representing 1.5×10^8^ to 1.5×10^−6^ molecules/µl.

**Figure 4:**
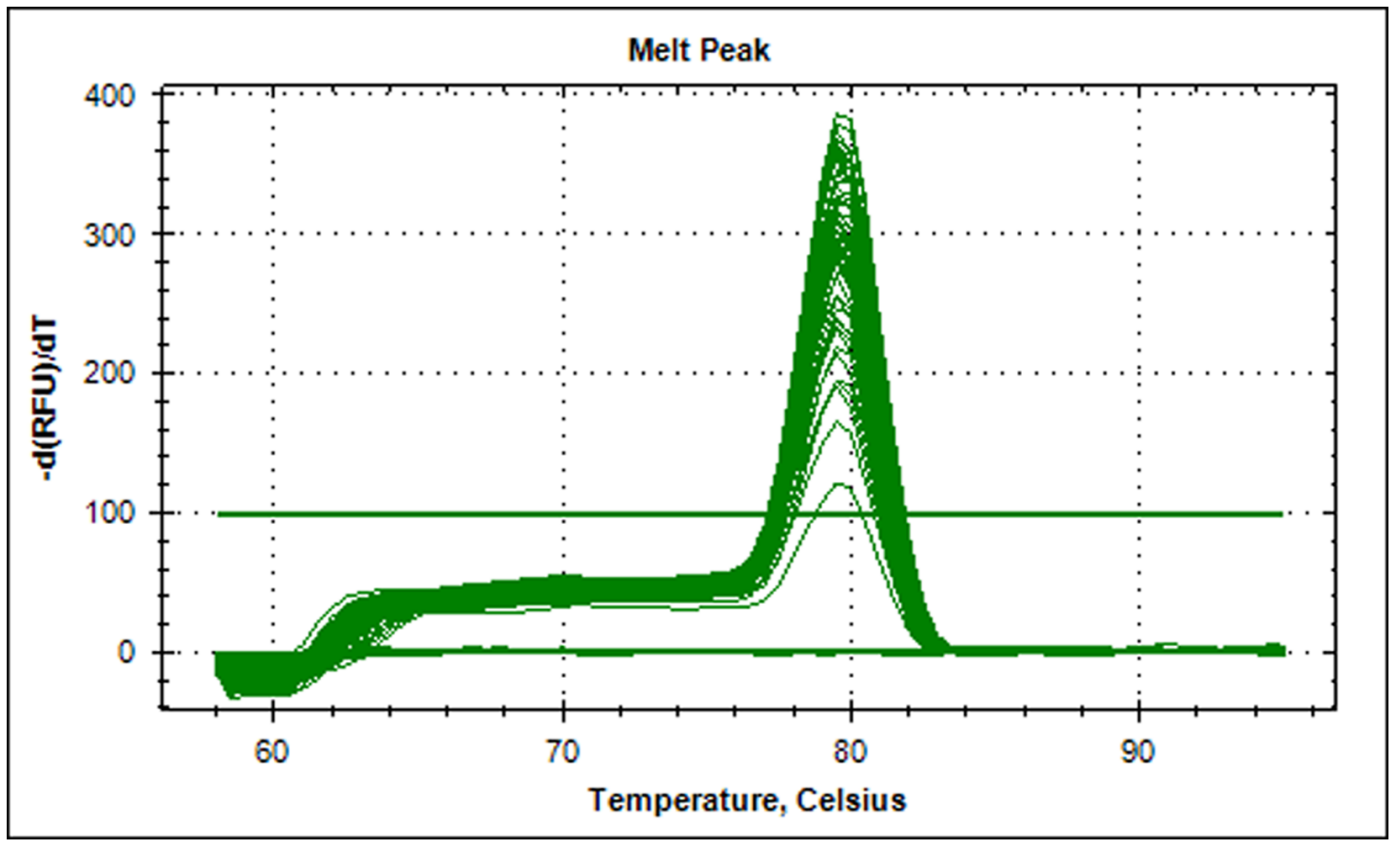
Melt curve analysis of PCR products.

Standard curves generated by the software were analyzed. A rise in Cq value was associated with a decrease in DNA content, pointing to a strong linear relationship between fluorescence and target copy number. The reaction was highly reproducible over a range of 10^8^ to a single copy number of the plasmid DNA, with a correlation coefficient (r^2^) of 0.984 and 99.0% efficiency (Fig. 3). No Cq value was observed for NAC and NTC. The assay detected as low as one plasmid molecule/µl of DNA. Amplified products were also subjected to melt curve analysis to confirm the presence of a single PCR product. Neither primer-dimer nor non-specific amplification products were observed on melt curve analysis (Fig. 4) or by gel electrophoresis (results not shown).

### Testing of Faecal Samples Collected from Naturally Infected Cattle and Buffaloes

**Figure 5:**
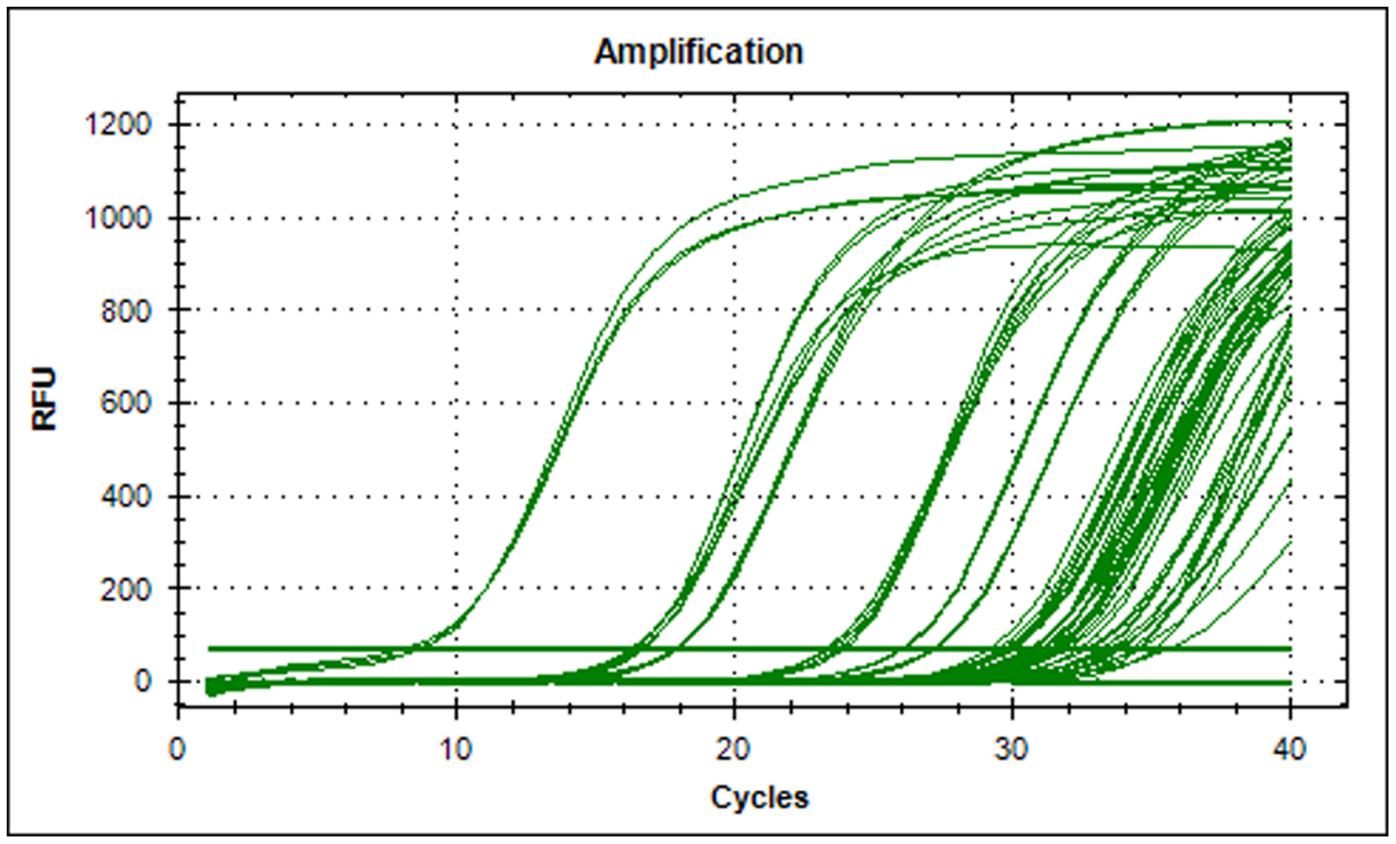
Fluorescence profile for standards and clinical samples used in real-time PCR assay.

Out of the total 65 samples screened, 41 were found positive for *M. digitatus* by the assay. The positive samples were quantified by the system based on the standard curve generated. The amplification plot for standard and unknown samples is shown in Fig. 5. The quantity of DNA in those samples ranged from 2.387 to 7.70×10^4^ molecules/µl (Table S1).

## Discussion

To date, there is no study reporting the use of real-time PCR for the detection of *M. digitatus*. Advances in nucleic acid testing offer more efficient and reliable methods like PCR-based assays, which have been used for detecting various parasites that affect livestock [18], [24]. The sequences of ITS-1 and ITS-2 of rDNA have been used extensively as a genetic marker for diagnostic purposes across a diverse range of organisms [19], [25], [26], [27], [28]. This study was undertaken to amplify the partial ITS-1 sequence in rDNA of *M. digitatus* using SYBR green as the fluorophore. The 182 bp PCR product was sequenced to confirm *M. digitatus*, and no amplification was observed with DNA from eight closely related species, i.e., *H. contortus*, *Trichostrongylus spp.*, *O. ostertagi*, *Oesophagostomum radiatum*, *Cooperia spp*., *Nematodirus spp.*, *Bunostomum spp.* and *Trichuris spp*. The conventional PCR detects 150 plasmids, whereas real-time PCR detects as low as one plasmid. In comparison to conventional PCR, the real-time assay was found to be sensitive and specific. Moreover, an evaluation of our real-time PCR assay using faecal DNA from cattle (n = 40) and buffaloes (n = 25) with naturally acquired trichostrongylid infections showed a significant correlation between Cq values and DNA amounts. In 2009, Learmount *et al*. [21] validated a qPCR assay for diagnosis of sheep nematodes *T. circumcincta* and *H. contortus* by harvesting eggs from the faecal samples. Whereas, in our study, we validated our qPCR assay by using the DNA extracted directly from faeces. The assay was good enough to produce reproducible results to detect the infection directly from faeces, thus reducing the time (3–5 days) needed for harvesting eggs by coproculture.

Currently, there is limited information available on the status of *M. digitatus* infection and its effect on the growth and reproductive parameters of cattle and buffaloes. This underpins the need for early and specific diagnostic assays. The present quantitative PCR-based assay was found to be specific and sensitive for the detection of *M. digitatus* from faecal samples. However, more such studies are needed in other countries to confirm the present finding. In addition to this, the real-time PCR assay can be used for large-scale epidemiological and population biology based studies [19], [29]. In conclusion, the developed qPCR assay will be useful to enhance our understanding on the severity of *M. digitatus* infection in large ruminants which in turn will be helpful in its treatment and control programs.

## Supporting Information

Table S1 **Validation of qPCR by testing faecal samples collected from cattle and buffaloes.** The Cq value and copies per µl of standard and unknown samples are mean values of the triplicates N/A- Not Applicable; *Tm-* Melting temperature.
(DOC)
