# Correction: Development and Validation of Real-Time PCR for Rapid Detection of *Mecistocirrus digitatus*

**DOI:** 10.1371/journal.pone.0113941

**Published:** 2014-11-12

**Authors:** The PLOS ONE Staff

The first two authors, Subhra Subhadra and Mohanraj Karthik, should be noted as contributing equally to this work.
