# Supplementary material for: Development and Validation of Real-Time PCR for Rapid Detection of Mecistocirrus digitatus
Source: PLoS One. 2013 Apr 30;8(4):e63019. doi: 10.1371/journal.pone.0063019 (PMC3639944; doi:10.1371/journal.pone.0063019)
Supplement: Table S1 — Validation of qPCR by testing faecal samples collected from cattle and buffaloes. The Cq value and copies per µl of standard and unknown samples are mean values of the triplicates N/A- Not Applicable; Tm- Melting temperature. (DOC) [file pone.0063019.s001.doc]

**Table S1: Validation of qPCR by testing faecal samples collected from cattle and buffaloes**

| **Sl. No.** | **Source of DNA** | **Content** | **Mean Cq** | **Mean Copies / µl** | ***T*m** | **Inference** |
| --- | --- | --- | --- | --- | --- | --- |
| 1 | Plasmid dilution 1 | Standard 1 | 8.51 | 1.50E+08 | 80.00 | Positive |
| 2 | Plasmid dilution 2 | Standard 2 | 16.45 | 1.50E+06 | 80.00 | Positive |
| 3 | Plasmid dilution 3 | Standard 3 | 23.84 | 1.50E+04 | 80.00 | Positive |
| 4 | Plasmid dilution 4 | Standard 4 | 31.10 | 1.50E+02 | 79.50 | Positive |
| 5 | Plasmid dilution 5 | Standard 5 | 34.64 | 1.50E+00 | 79.50 | Positive |
| 6 | Plasmid dilution 6 | Standard 6 | N/A | 1.50E-02 | None | Negative |
| 7 | Plasmid dilution 7 | Standard 7 | N/A | 1.50E-04 | None | Negative |
| 8 | Plasmid dilution 8 | Standard 8 | N/A | 1.50E-06 | None | Negative |
| 9 | *M. digitatus* genomic DNA | Positive | 16.51 | 1.23E+06 | 79.50 | Positive |
| 10 | *M. digitatus* genomic DNA | Positive | 17.94 | 4.58E+05 | 79.50 | Positive |
| 11 | Faecal DNA – Cattle (NDC) | Unknown | N/A | N/A | None | Negative |
| 12 | Faecal DNA – Cattle (NDC) | Unknown | 29.47 | 1.66E+02 | 79.50 | Positive |
| 13 | Faecal DNA – Cattle (NDC) | Unknown | N/A | N/A | None | Negative |
| 14 | Faecal DNA – Cattle (NDC) | Unknown | 34.61 | 5.50E+00 | 79.50 | Positive |
| 15 | Faecal DNA – Cattle (NDC) | Unknown | N/A | N/A | None | Negative |
| 16 | Faecal DNA – Cattle (NDC) | Unknown | N/A | N/A | None | Negative |
| 17 | Faecal DNA – Cattle (NDC) | Unknown | 23.78 | 8.25E+03 | 79.50 | Positive |
| 18 | Faecal DNA - Cattle (NDC) | Unknown | 34.45 | 5.33E+00 | 79.50 | Positive |
| 19 | Faecal DNA - Cattle (NDC) | Unknown | 30.73 | 6.97E+01 | 79.50 | Positive |
| 20 | Faecal DNA - Cattle (NDC) | Unknown | N/A | N/A | None | Negative |
| 21 | Faecal DNA - Cattle (NDC) | Unknown | N/A | N/A | None | Negative |
| 22 | Faecal DNA - Cattle (NDC) | Unknown | 27.17 | 8.00E+02 | 79.50 | Positive |
| 23 | Faecal DNA - Cattle (NDC) | Unknown | 33.34 | 1.22E+01 | 79.50 | Positive |
| 24 | Faecal DNA - Cattle (NDC) | Unknown | 31.57 | 3.88E+01 | 79.50 | Positive |
| 25 | Faecal DNA - Cattle (NDC) | Unknown | 30.85 | 6.43E+01 | 79.50 | Positive |
| 26 | Faecal DNA - Cattle (NDC) | Unknown | 33.48 | 1.07E+01 | 79.50 | Positive |
| 27 | Faecal DNA - Cattle (NDC) | Unknown | 31.82 | 3.29E+01 | 79.50 | Positive |
| 28 | Faecal DNA - Cattle (NDC) | Unknown | 23.37 | 1.10E+04 | 79.50 | Positive |
| 29 | Faecal DNA - Cattle (NDC) | Unknown | 31.84 | 3.35E+01 | 79.50 | Positive |
| 30 | Faecal DNA - Cattle (NDC) | Unknown | 31.61 | 3.78E+01 | 79.50 | Positive |
| 31 | Faecal DNA - Cattle (NDC) | Unknown | N/A | N/A | None | Negative |
| 32 | Faecal DNA - Cattle (NDC) | Unknown | N/A | N/A | None | Negative |
| 33 | Faecal DNA - Cattle (NDC) | Unknown | 27.51 | 6.33E+02 | 79.50 | Positive |
| 34 | Faecal DNA - Cattle (CBJC) | Unknown | 15.20 | 3.03E+06 | 79.50 | Positive |
| 35 | Faecal DNA - Cattle (CBJC) | Unknown | N/A | N/A | None | Negative |
| 36 | Faecal DNA - Cattle (CBJC) | Unknown | 33.53 | 1.00E+01 | 79.50 | Positive |
| 37 | Faecal DNA - Cattle (CBJC) | Unknown | N/A | N/A | None | Negative |
| 38 | Faecal DNA - Cattle (CBJC) | Unknown | 28.37 | 3.50E+02 | 79.50 | Positive |
| 39 | Faecal DNA - Cattle (CBJC) | Unknown | 13.1 | 1.28E+07 | 79.50 | Positive |
| 40 | Faecal DNA - Cattle (CBJC) | Unknown | 30.60 | 7.55E+00 | 79.50 | Positive |
| 41 | Faecal DNA - Cattle (CBHFC) | Unknown | N/A | N/A | None | Negative |
| 42 | Faecal DNA - Cattle (CBHFC) | Unknown | N/A | N/A | None | Negative |
| 43 | Faecal DNA - Cattle (CBHFC) | Unknown | 33.61 | 9.50E+00 | 79.50 | Positive |
| 44 | Faecal DNA - Cattle (CBHFC) | Unknown | 26.62 | 1.17E+03 | 79.50 | Positive |
| 45 | Faecal DNA - Cattle (CBHFC) | Unknown | N/A | N/A | None | Negative |
| 46 | Faecal DNA - Cattle (CBHFC) | Unknown | 27.02 | 8.87E+02 | 79.50 | Positive |
| 47 | Faecal DNA - Cattle (CBHFC) | Unknown | 26.31 | 1.45E+03 | 79.50 | Positive |
| 48 | Faecal DNA - Cattle (CBHFC) | Unknown | N/A | N/A | None | Negative |
| 49 | Faecal DNA - Cattle (CBHFC) | Unknown | N/A | N/A | None | Negative |
| 50 | Faecal DNA - Cattle (CBHFC) | Unknown | N/A | N/A | None | Negative |
| 51 | Faecal DNA – Buffalo (NDB) | Unknown | 31.48 | 4.90E+01 | 79.50 | Positive |
| 52 | Faecal DNA - Buffalo (NDB) | Unknown | 29.95 | 1.18E+02 | 79.50 | Positive |
| 53 | Faecal DNA - Buffalo (NDB) | Unknown | 29.87 | 1.25E+02 | 79.50 | Positive |
| 54 | Faecal DNA - Buffalo (NDB) | Unknown | 26.06 | 1.72E+03 | 79.50 | Positive |
| 55 | Faecal DNA - Buffalo (NDB) | Unknown | N/A | N/A | None | Negative |
| 56 | Faecal DNA - Buffalo (NDB) | Unknown | N/A | N/A | None | Negative |
| 57 | Faecal DNA - Buffalo (NDB) | Unknown | 25.95 | 1.85E+03 | 79.50 | Positive |
| 58 | Faecal DNA - Buffalo (NDB) | Unknown | 23.01 | 1.40E+04 | 79.50 | Positive |
| 59 | Faecal DNA - Buffalo (NDB) | Unknown | 27.85 | 5.01E+02 | 79.50 | Positive |
| 60 | Faecal DNA - Buffalo (NDB) | Unknown | N/A | N/A | None | Negative |
| 61 | Faecal DNA - Buffalo (NDB) | Unknown | 26.55 | 1.22E+03 | 79.50 | Positive |
| 62 | Faecal DNA - Buffalo (NDB) | Unknown | 28.71 | 2.77E+02 | 79.50 | Positive |
| 63 | Faecal DNA - Buffalo (NDB) | Unknown | 27.51 | 6.33E+02 | 79.50 | Positive |
| 64 | Faecal DNA - Buffalo (NDB) | Unknown | N/A | N/A | None | Negative |
| 65 | Faecal DNA - Buffalo (NDB) | Unknown | 27.55 | 6.15E+02 | 79.50 | Positive |
| 66 | Faecal DNA - Buffalo (NDB) | Unknown | N/A | N/A | None | Negative |
| 67 | Faecal DNA - Buffalo (NDB) | Unknown | N/A | N/A | None | Negative |
| 68 | Faecal DNA - Buffalo (NDB) | Unknown | 26.18 | 1.58E+03 | 79.50 | Positive |
| 69 | Faecal DNA - Buffalo (GMB) | Unknown | 28.18 | 3.99E+02 | 79.50 | Positive |
| 70 | Faecal DNA - Buffalo (GMB) | Unknown | 28.41 | 3.40E+02 | 79.50 | Positive |
| 71 | Faecal DNA - Buffalo (GMB) | Unknown | N/A | N/A | None | Negative |
| 72 | Faecal DNA - Buffalo (GMB) | Unknown | 25.69 | 2.21E+03 | 79.50 | Positive |
| 73 | Faecal DNA - Buffalo (GMB) | Unknown | 27.97 | 4.61E+02 | 79.50 | Positive |
| 74 | Faecal DNA - Buffalo (GMB) | Unknown | N/A | N/A | None | Negative |
| 75 | Faecal DNA - Buffalo (GMB) | Unknown | 28.21 | 3.91E+02 | 79.50 | Positive |
| 76 | *H. contortus* genomic DNA | NAC | N/A | N/A | None | Negative |
| 77 | No template control | NTC | N/A | N/A | None | Negative |

The presented Cq value and copies per µl of standard and unknown samples are mean values of the triplicates

N/A – Not Applicable; *Tm* – Melting temperature
